# A review: a comparison of different adsorbents for removal of Cr (VI), Cd (II) and Ni (II)

**DOI:** 10.3906/kim-2002-21

**Published:** 2020-08-18

**Authors:** Anja PFEIFER, Mojca ŠKERGET

**Affiliations:** 1 Faculty of Chemistry and Chemical Engineering, University of Maribor, Maribor Slovenia

**Keywords:** Adsorption, chromium, cadmium, nickel, adsorption comparison

## Abstract

A review of the studies dealing with the removal of chromium, cadmium, and nickel ions with different adsorbents published in the literature between 2014 and 2018 is given in tabular form, along with the adsorption conditions, adsorption isotherm, and kinetic models applied by the authors to model the experimental data and adsorption capacities. The review focuses on the efficiency of ion removal.

## 1. Introduction

The evolution of civilization has led to the growth and development of all of the necessities that mankind needs for a comfortable life. Various industries have flourished (chemical, metallurgical, food, pharmaceutical, agricultural industry, etc.), which has resulted in environmental pollution.

According to the Reference Document for Common Waste Water and Waste Gas Treatment Data for Europe (EU-27 plus Iceland, Liechtenstein, Norway, and Serbia), water is contaminated annually with 18.53 tons of cadmium, 284.4 tons of chromium, and 324.9 tons of nickel [1]. These metals are problematic because of their toxicity; their emissions must therefore be kept under control.

With growing awareness of the consequences, legislation has become stricter; much effort has been invested in developing procedures for establishing and maintaining a cleaner environment.

The most common procedures for the removal of pollutants from wastewater and industry effluents are ion exchange, membrane separation processes (reverse oS_M_osis, electrolysis), evaporation, chemical precipitation, reduction, coagulation, flocculation, solvent extraction, photo reduction, and adsorption [2,3]. Membrane separation processes and evaporation are quite expensive, as they use much energy. For optimal procedure conditions and for enhancing all of the above-stated operations, many additional reagents are used. Removal procedures usually produce secondary wastes, which are usually quite concentrated and hard to discard or recycle [4].

Many studies focus on adsorption. Commercial adsorbents are available, but most of them are expensive and difficult to regenerate and separate from the rest of the solution; furthermore, they lack selectivity and adsorption capacity. The development of adsorbents is focused now on alternative sources; researchers are focusing on utilizing biomass residue, which is abundantly available throughout the world [5,6].

### 1.1. Adsorbents and adsorption

Adsorption is the process wherein components from gas or liquid phases bind to the surface of a solid substance. Binding itself can occur on a physical or chemical basis. Adsorbents are usually porous materials and usually have a large surface area due to their high porosity. Because of porosity, the internal area is generally bigger than the external area [7].

Adsorbents can be divided into 3 groups: natural inorganic, natural organic, and synthetic adsorbents. Natural inorganic adsorbents are clays, minerals (e.g., goethite, hydroxy-apatite, calcite), fly ash, zeolite, calcareous soils, slags, sludges, modified asphaltite, etc. Natural organic adsorbents include sawdust, coconut shell, corncob waste, tea waste, rice hulls, bark, hazelnut shells, wool, peat, and chitosan; synthetic adsorbents include nano-sized metal oxides, zero-valent iron, modified nano materials, etc. Nanoadsorbents, especially magnetic nanoadsorbents, have enormous industrial potential due to their high reactivity, many active sites, and consequent large surface area. Their shortcomings include instability and consequent aggregation, which diminishes their surface area; as a result, their reactivity is reduced. For prevention of aggregation and dissolvement, different additives like clays and polymers are generally used [8,9].

Due to the circular economy, natural organic and inorganic adsorbents have tremendous potential for general use. Many residues and wastes from industrial and agricultural activities are used. In order to optimize their functionality and properties, adsorbents can be modified and improved with different functional groups, which can provide better results in pollutant removal.

Many factors can influence the course of adsorption. For example, pH value has tremendous influence on adsorption’s course, as it affects the chemical state of the solution as well as the chemical state of the adsorbent and its surface [10]. With a pH value of zero-point charge (pH_pzc_) and with a charge of adsorbate, it is possible to estimate which pH value of the solution would be appropriate for the removal of a particular adsorbate. If the pH value is below the pH value of zero-point charge, the adsorbent surface is positively charged and attracts anions; if the pH value is above pH_pzc_, the surface has a negative charge and attracts cations [11]. It is therefore necessary to know the chemistry of the adsorbate itself. At specific pH values, precipitation can occur, and the adsorption results may therefore be faulty.

The course of adsorption can also be altered and influenced by changing the initial concentration of adsorbate or by changing the dose of the adsorbent. Both variables can increase or decrease adsorption. In some cases, a higher initial concentration shows a large increase in adsorption rate, because it creates the necessary driving force to overcome liquid resistance. The rate of adsorption may decrease quickly after initial adsorption, because the active sites that are easily accessible are occupied, causing a slight change in the adsorbent surface’s charge. Because easily accessible places are already occupied, only places that are difficult to access remain. Occupied sites also influence the surface charge, which results in a decrease in the adsorption rate [9,12].

A higher adsorbent dose generates more active sites for binding; thus, adsorption rate usually increases to some extent. If the adsorbent dose is too high, it can diminish adsorption rate, because aggregates are formed and the number of active sites decreases [13].

## 2. Review of adsorbents for chromium, cadmium, and nickel removal

### 2.1. Chromium

Chromium is the thirteenth most common element found in the earth’s crust. It is usually found in ore chromate. Chromium and its compounds are used in the manufacture of stainless steel, alloys, metal plating and finishing 860 solutions, leather tanning, pigment, and wood preservatives; it can be used as a corrosion inhibitor and as a catalyst. It has many oxidation states, but the most toxic form is hexavalent chromium. It causes severe health problems, especially due to its oxidating properties and its high solubility in water, which makes it available for biological uptake [14,15].

Cr (VI) likes to bond with hydroxyl groups; in most studies, the adsorption of Cr (VI) was successful when pH value was rather low, especially if pH value was below pH_pzc_. With a pH value below pH_pzc_, Cr (VI) is pulled toward the negatively charged surface of the adsorbent. If the pH value is above pH_pzc_, chromium ions are repelled by the adsorbent surface and adsorption is decreased. The adsorption selectivity at high and low pH values can be explained by different forms of chromium in the aqueous environment. At pH values below 1, chromium exists as H_2_CrO_4_ . When the pH value is between 2 and 6.0, the dominant species HCrO^-^_4_ and Cr_2_O^2-^_7_ form; with pH values above 6.0, CrO^2-^_4_ is dominant, which is harder to bind [10,15].

Most of the reviewed experiments were conducted at pH value 3. In some studies, experiments were performed at higher pH values primarily to prevent the adsorbent from dissolving. Higher pH values (above 6) will also cause chromium precipitation.

By increasing the initial Cr (VI) concentration and maintaining the adsorbent dose, the percentage of adsorbed Cr (VI) may be lower because the number of active sites is limited; when they are occupied, adsorption is complete. In addition, the reason for lower adsorption may be found in the formation of a film around the adsorbent, which prevents the adsorbate from accessing the surface. At the same time, the adsorbent capacity may be higher, because the higher initial concentration provides the necessary driving force to overcome liquid resistance [8,16].

Depending on the adsorbent, a higher adsorbent dose will increase adsorption because of the higher number of active sites. If the adsorption is slower, the main cause is the aggregation of adsorbent particles, which resultes in fewer active sites [8].

The overview of the adsorption studies of chromium (VI) is summarized in Table 1.

**Table 1:** Chromium (VI) adsorption capacities/parameters.

Adsorbent	Adsorbent characterization	Adsorption conditions	Isotherm and kinetic models with calculated constants and adsorption capacities				Adsorption capacity	Ref
			Isotherm model	Calculated constants	Kinetic model	Calculated constants	Experimental	
Synthetic adsorbents								
Fe_3_O_4_-NH_2_ (amino functionalized magnetic nanoadsorbent	D_50_ = 25 nm, pH_pzc_ = 5.8	pH = 3, T = 298 K, c_0_ = 5 mg/L, m_ads_/V = 0.05 g/50 mL	Langmuir	q_m_ = 232.51 mg/g	Pseudosecond	R^2^ = 0.9871 q_e,cal_ = 28.25 mg/g	R% > 96 % q_e,exp_ = 24.25 mg/g	[2]
Ppy-Fe_3_O_4_/rGO (magnetic composite of reduced graphene oxide, polypirrole and Fe_3_O_4_ nano particles	S_M_ = 80.53 m^2^/g	pH = 3, T = 318 K, c_0_ = 48.4 mg/L, m_ads_/V = 0.25 g/L	Langmuir	q_m_ = 293.3 mg/g	/	/	/	[10]
Magnetite nanoparticles	D_50_ = 56 nm	pH = 3, T = 30 ◦C	Freundlich	R^2^ = 0.996 K_f_ = 3.81 mg/g	/	/	R% = 72 %	[13]
EDA-MPMs (ethylenediamine functionalized magnetic polymer microspheres)	S_M_ = 286 m^2^/g, V_P_ = 0.512 cm^3^/g, D_P_ = 6.8 nm	pH = 2, T = 298 K, c_0_ = 300 mg/L, m_ads_/V = 0.05 g/50 mL	Langmuir	R^2^ = 0.999 q_m_ = 236.9 mg/g	Pseudo second	R^2^ = 0.997 q_e,cal_ = 169.205 mg/g	q_e,exp_ = 145.2 mg/g	[15]
MWCNT (magnetic multiwall carbon nanotubes)	S_M_ = 200 m^2^/g, D_50_ = 20–40 nm, L = 2 μm	pH = 3, c_0_ = 2 mg/L, m_ads_/V = 0.08 g/100 mL	Langmuir	R^2^ = 0.998 q_m_ = 16.234 mg/g	Pseudosecond	R^2^ = 0.9997 q_e,cal_ = 1.747 mg/g	R% = 100 % q_e,exp_ = 1.729 mg/g	[17]
Nanomagnetite particles (low pressure synthetic procedure)	/	pH = 4, c_0_ = 0,1 mg/L, m_ads_/V = 10 mg/4 mL, t_cont._ = 1 h	Langmuir	R^2^ = 0.995	/	/	R% = 100 % q_e,exp_ = 1.705 mg/g	[18]
BaFe_12_O_19_ magnetic nanopowder (sample 4)	S_M_ = 13 m^2^/g, D = 87 nm, pH_pzc_ = 5 ± 0.5	pH = 3, T = 25 ◦C, c_0_ = 133 mg/L, m_ads_/V = 1.5 g/150mL, t_cont._ = 1 h	/	/	Pseudosecond	R^2^ = 0.9706 q_e,cal_ = 14.3 mg/g	R% = 99.5 % q_e,exp_ = 13.25 mg/g	[19]
IL-oxi-MWCNT (ionic liquid functionalized oxidized multiwalled carbon nanotubes)	S_M_ = 87.4 m^2^/g, V_P_ = 0.82 cm^3^/g, pH_pzc_ = 4–2	pH = 2.5–4, c_0_ = 20 mg/L, m_ads_/V = 0.15 g/20 mL	Langmuir	R^2^ = 0.96 q_m_ = 85.83 mg/g	Pseudosecond	R^2^ = 0.91 q_e,cal_ = 2.58 mg/g	R% = 99.5 % q_e,exp_ = 2.54 mg/g	[20]
Fe_3_O_4_ nanoparticles	S_M_ = 42.53 m^2^/g, d_50_ = 15 nm	pH = 3, T = 55 ◦C, c_0_ = 10 mg/L, m_ads_ = 0.5 g/L, t_cont._ = 90 min	/	/	/	/	R% = 88 % q_e,exp_ = 33.45 mg/g	[37]
Fe_3_O_4_/CTAB (Fe_3_O_4_ nanoparticles caped with cetyltrimethylammonium bromide)	Fe_3_O_4_ crystal: D_50_ = 16.25 nm	pH = 4, T = 25 ± 1 ◦C, c_0_ = 100 mg/L, m_ads_/V = 12 mg/mL, t_cont._ = 12 h	Langmuir	R^2^ = 0.99, q_m_ = 18.5 mg/g	Pseudosecond	R^2^ = 0.99	R% = 95.77 %q _e,exp_ = 10.05 mg/g	[38]
PANISA (Polianiline nanofibers; PANI; acid treated natural silica)	PANI nanofibers: D = 30–80 nm	pH = 2, T = 25 ◦C, c_0_ = 20 mg/L, m_ads_/V = 4 g/L, t_cont._. = 2 min	/	/	/	/	R% = 99.31 %	[39]
WT (NH_3_) Magnetic nanocomposite (from waste toner)	S_M_ = 42.53 m^2^/g, D_50_ = 53.5 nm	pH = 2, T = 25 ◦C, c_0_ = 50 mg/L, m_ads_/V = 4 g/L	Langmuir	/	Pseudosecond	/	R% = 99 %	[40]
Core-shelled Fe_3_O_4_ hybrid nanoparticle aggregate	S_M_ = 238.18 m^2^/g, D_50_ = 700 nm, D_P50_ = 7.5–9.1 nm	pH = 3, c_0_ = 10 mg/L, m_ads_/V = 20 mg/50 mL	/	/	/	/	R% = 94.8 %	[41]
h.mag-poly (EGDMA-VIM) microbeads	S_M_ = 81.4 m^2^/g D = 53–211 μm	pH = 2, c_0_ = 50 mg/L, m_ads_/V = 50 mg/50 mL	Langmuir	R^2^ = 0.995 q_m_ = 174.3 mg/g	/	/	R% = 32 % q_e,exp_ = 16.2 mg/g	[42]
Natural organic/inorganic adsorbents								
Grape waste	D_50_ = 0.2–0.5 mm	pH = 2, T = 20 ◦C, c_0_ = 120 mg/L, m_ads_/V = 0.05 g/100 mL	Langmuir	R^2^ = 0.9847 q_m_ = 108.12 mg/g	Pseudosecond	R^2^ = 0.997 q_e,cal_ = 104.5	q_e,exp_ = 104.3 mg/g	[4]
FBA-Fe/Ni NP (Fe/Ni nanoparticles supported by a novel fly ash-based porous adsorbent)	S_M_ = 20.63 m^2^/g, D_P50_ = 2.89 nm	pH = 1, T = 303 K, c_0_ = 1000 mg/L, m_ads_/V = 0.2 g/100 ml, t_cont._ = 24 h	/	/	/	/	R% < 6 % q_e,exp_ = 25.07 mg/g	[8]
BCS (biochar from wheat straw)	S_M_ = 26.3 m^2^/g, V_P_ = 0.026 cm^3^/g	pH = 2, T = 25 ◦C, c_0_ = 100 mg/L, m_ads_/V = 0.2 g/50 mL, t_cont._ = 21 h	Langmuir	R^2^ = 0.990 q_m_ = 24.6 mg/g	Pseudosecond	R^2^ = 0.983 q_e,exp_ = 14.36 mg/g	q_e,exp_ = 14.36 mg/g	[14]
BCW (biochar from whicker)	S_M_ = 11.4 m^2^/g V_P_ = 0.0061 cm^3^/g	pH = 2, T = 25 ◦C, c_0_ = 100 mg/L, m_ads_/V = 0.2 g/50 mL, t_cont._ = 18 h	Langmuir	R^2^ = 0.978 q_m_ = 23.6 mg/g	Pseudosecond	R^2^ = 0.992 q_e,cal_ = 10.31 mg/g	q_e,exp_ = 10.28 mg/g	[14]
Andisols clayzeolite composite	S_M_ = 48.61 m^2^/g, D_P_ = 95.9 Å	pH = 4, c_0_ = 2 mg/L, m_ads_/V = 0.1 g/10 mL, t_cont._ = 60 min	Freundlich	R^2^ = 0.963 K_f_ = 0.17 mg/g	/	/	R% = 76.10 %q_e,exp_ = 0.16 mg/g	[16]
Microporous activated carbon from almond shell	S_M_ = 1223 m^2^/g, V_P_ = 0.326 cm^2^/g, D_P50_ = 2.39 nm, pH_pzc_ = 5.5	pH = 2, T = 25 ◦C, c_0_ = 100 mg/L, m_ads_/V = 2.5 g/L, t_cont._ = 240 min	Langmuir	R^2^ = 0.976 q_m_ = 195.34 mg/g	Pseudosecond	R^2^ = 0.999 q_e,cal_ = 47.6 mg/g	R% = 100 %, q_e,exp_ = 45.12 mg/g	[21]
FNAC (activated carbon from fox nutshell)	S_M_ = 2869 m^2^/g, V_P_ = 1.96 cm^3^/g	pH = 2, T = 30 ◦C, c_0_ = 10 mg/L, m_ads_/V = 0.05 g/100 ml, t_cont._ = 3 h	Langmuir	R^2^ = 0.971, q_m_ = 46.21 mg/g	Pseudosecond	R^2^ = 0.999, q_e,cal_ = 43.99 mg/g	R% = 99.08 %, q_e,exp_ = 43.45 mg/g	[22]
Modified activated carbon	S_M_ = 1.4 m^2^/g	pH = 3, c_0_ = 1 mg/L, m_ads_ = 75 mg, t_cont._ = 4 h	Langmuir	R^2^ = 0.94 q_m_ = 18.519 mg/g	Pseudosecond	R^2^ = 0.994 q_e,cal_ = 1.805 mg/g	R% = 99 %	[23]
GSC (Graphene sand composite)	S_M_ = 157 m^2^/g	pH = 1.5, c_0_ = 20 mg/L	Langmuir	R^2^ = 0.99108, q_m_ = 2859.38 mg/g	Pseudosecond	R^2^ = 0.98003	R% = 93 %	[24]
CMAC (Activated carbon from Cucumis melo peel)		pH = 3, c_0_ = 100 mg/L, m_ads_ = 250 mg/L, t_cont._ = 180 min	Elovich	R^2^ = 0.978	Pseudofirst	R_2_ = 0.996	R% = 97.95 %q_e,exp_ = 7.29 mg/g	[34]
La-RM (red mud modified by lanthanum)	D_50_ = 25.6 nm	pH = 7, T = 25 ◦C, c_0_ = 40 mg/L, m_ads_/V = 4 g/L	Langmuir	R^2^ = 0.9956, q_m_ = 16.581 mg/g	Pseudosecond	/	q_e,exp_ = 17.35 mg/g	[36]
ACLL (Activated carbon from leucaena leucocepnala)	S_M_ = 1131 m^2^/g, d_50_ = 250 μm, pH_pzc_ = 5.42	pH = 4, T = 30 ◦C, c_0_ = 100 mg/L, m_ads_/V = 300 mg/50 mL, t_cant._ = 60 min	Redlich-Peterson	R^2^ = 0.994	Pseudosecond	R^2^ = 0.99 q_e,cal_ = 14.29 mg/g	R% < 80 % q_e,exp_ = 13.85 mg/g	[43]
20 wt% β-FeOOH/SYBK (activated carbon suported β-FeOOH)	S_M_ = 670.65 m^2^/g, D_P50_ = 2.66 nm, V_P_ = 0.447 cm^3^/g	pH = 2, c_0_ = 49.21 mg/L, m_ads_/V = 2 g/L, t_cont._ = 60 min	Langmuir	R^2^ = 0.9799, q_m_ = 37.036 g/kg	Pseudofirst	R^2^ = 0.975	R% = 96% q_e,exp_ = 37.04 g/kg	[44]
WPAC (wood based activated carbon)	S_M_ > 1000 m^2^/g	pH = 3, T = 25 ◦C, c_0_ = 80 mg/L, m_ads_/V = 20 mg/50 mL, t_cont._ = 24 h	/	/	/	/	R% = 40.04 %q_e,exp_ = 70.95 mg/g	[45]
BCP (biochar Onopordom Heteracanthom)	S_M_ = 5.73 m^2^/g D_P_ = 1–50 nm, pH_pzc_ = 2	pH = 2, T = 15 ± 1 ◦C, c_0_ = 40 mg/L, m_ads_/V = 0.1 g/100 mL	Langmuir	R^2^ = 0.9795, q_m_ = 37.28 mg/g	Pseudosecond	R^2^ = 0.994 q_e,cal_ = 31.08 mg/g	R% = 69 %	[46]
Chitosanmagnetite nanocomposite strip	D_50_ = 15–30 nm	pH = 2–3, t_cont._ = 130 min	/	/	/	/	R%=92.33 %	[47]
Algae biomass from C.glomerata		pH = 2, T = 45 ◦C, c_0_ = 20 mg/L, m_ads_/V = 1 g/100 mL, t_c_ = 60 min	Freundlich	R^2^ = 0.949, K_f_ = 0.19 mg/g	/	/	R% = 66.6 %	[48]
Kaolin	S_M_ = 14.93 ± 0.32 m^2^/g, V_P_ = 0.167 ± 0.27 cm^3^/g, D_P_ = 12.59 ± 0.49 nm	pH = 4.5, T = 25 ± 1◦C, c_0_ = 200 mg/L, m_ads_/V = 0.5 g/20 mL, t_cont._ = 120 min	Langmuir	R_2_ = 0.985 q_m_ = 0.878 mg/g	Pseudofirst	R^2^ = 0.993 q_e,cal_ = 0.865 mg/g	q_e,exp_ = 0.853 mg/g	[49]
MCARM (mechanicalchemical activated red mud)	S_M_ = 176 m^2^/g D_50_ = 3.83 μm	pH = 2, c_0_ = 400 mg/L, m_ads_ = 4.8 g	Langmuir	R^2^ = .9903 q_m_ = 6.7 mg/g	Pseudosecond	R^2^ = 0.999	R% = 95 % q_e,exp_ = 5.45 mg/g	[50]
ARM (heat-acid active red mud)	pH_pzc_ = 8.5	pH = 2, T = 30 ◦C, c_0_ = 0.08 mg/L, m_ads_/V = 0.5 g/100 mL, t_c_ = 120 min	Langmuir	q_m_ = 0.03 mg/g	/	/	R% = 97.31 %q_e,exp_ = 0.015 mg/g	[51]
PPH (powdered Peganum harmalo)	S_M_ = 7.8 m^2^/g pH_pzc_ = 6.4	pH = 1.5, c_0_ = 100 mg/L, m_ads_/V = 10 g/L, t_cont._= 80 min	Freundlich	R^2^ = 0.986	Pseudosecond	R^2^ = 0.99 q_e,cal_ = 10.13 mg/g	q_e,exp_ = 9.99 mg/g	[52]
Fungal biomass		pH = 2, T = 27 ◦C, c_0_ = 62.5 mg/L, m_ads_/V = 2 g/L, t_cont._ = 37.5 min	Langmuir	R^2^2 = 0.999 q_m_ = 71.9 mg/g	Pseudosecond	R > 0.99	R% = 63.82 %	[53]
Biomass from Ceratocystis paradoxa MSR^2^	D = 80 μm	pH = 2, c_0_ = 62.5 mg/L, m_ads_/V = 2 g/L, t_cont._ = 60 min	Langmuir	R^2^ = 0.991 q_m_ = 72.46 mg/g	Pseudosecond	R^2^ > 99 %	R% = 68.72 %	[54]

D_50_ – average particle size, pH_pzc_ – point of zero charge, S_M_ – specific surface area in a unit of mass, V_P_ – pore volume, D_P_ – pore size, D – particle size, L – lenght, D_P50_– average pore size, c_0_ – initial concentration, m_ads_/V – adsorbent dose per sample volume, t_cont._ - the time of contact od solution and adsorbent, m_ads_ – adsorbent dose, q_m_ – maximum adsorbent capacity (Langmuir), K_f_ – adsorption capacity (Freundlich), q_e,cal_ – calculated adsorption capacity, q_e,exp_ – experimentally determined adsorption capacity, R% – adsorption percent.

A review of studies with synthetic adsorbents showed that 100% chromium removal was achieved with the use of magnetic multiwall carbon nanotubes (MWCNT) [17] and nanomagnetite particles, which were synthesized in a low-pressure procedure [18]. The use of BaFe_12_O_19_ magnetic nano powder [19] and ionic liquid functionalized oxidized multiwall carbon nanotubes (IL-oxi-MWCNT) resulted in 99.5% removal [20]. The maximum adsorbent capacity of 293.3 mg/g was determined with the use of a magnetic composite of reduced graphene oxide, polypirrole, and Fe_3_O_4_ nanoparticles (Ppy-Fe_3_O_4_/rGO) [10]. The surface area of Ppy-Fe_3_O_4_/rGO was 80 m^2^/g. In the same study, the comparison was made with Fe_3_O_4_/rGO. The adsorption capacity of Ppy-Fe_3_O_4_/rGO was higher, even though the surface area of Fe_3_O_4_/rGO was 126.42 m^2^/g. It was established that the surface area does not represent a decisive parameter in the adsorption process. Ppy-Fe_3_O_4_/rGO showed excellent adsorption properties. Chain-like Ppy forms a 3-dimensional network with Fe_3_O_4_ nanoparticles and with rGO sheets. The adsorption process happens because of electrostatic attraction, ion exchange, and chemical reduction of Cr (VI) to Cr (III) [10].

Similar adsorption capacities of 236.9 mg/g [15] and 232.51 mg/g [2] were determined with the use of ethylenediamine-functionalized magnetic polymer microspheres (EDA-MPMs) [15] and amino-functionalized magnetic nanoadsorbent (Fe_3_O_4_-NH_2_) [2], respectively. By increasing the temperature to 318 K, the EDAMPMs achieved a maximum adsorption capacity of 253.2 mg/g. Synthesis of EDA-MPM’s adsorbent starts in the presence of Fe_3_O_4_ nanoparticles. After polymerization, the particles are modified with ethylenediamine [15]. Through synthesis of Fe_3_O_4_-NH_2_ , amino groups were fixed to the surface of Fe_3_O_4_ nuclei and a core–shell structure was created. The core–shell structure is considered to have unique properties such as stability and chemical capability, which are important properties of the adsorbent [2]. Both adsorption procedures were conducted in an acidic environment and both adsorbents had similar chemistry behind their successful adsorption capacity. Because of the acidic conditions in which both experiments were conducted, the amino groups were protonated to NH^3+^ , resulting in an even stronger electrostatic attraction between NH^3+^ and Cr (VI) [2,15]. In the natural organic and inorganic adsorbent section, Cr (VI) removal of 100% [21], 99.08% [22], and 99% [23] was achieved with the use of microporous activated carbon from almond shell [21], activated carbon from fox shell (FNAC) [22], and with modified activated carbon [23]. Among the 3 adsorbents, microporous activated carbon had the highest maximum capacity of 195.34 mg/g [21].

Astonishing results were achieved with the graphene–sand composite (GSC) [24], where the maximum adsorption capacity calculated with the Langmuir model was 2859.38 mg/g. In the study, the high adsorption capacity was attributed to the graphene sheets on the sand surface used for preparation of the composite [24].

For the adsorbents mentioned above with the highest adsorption capacities, we found that the researchers proposed a similar binding mechaniS_M_ for chromium adsorption. Adsorption is more successful at low pH values, from pH 1 to 4. In this case, there are many H^+^ ions in the solution that protonate the surface of the adsorbent. At low pH levels, the chromium in the solution is in the form of HCrO^-^_4_ and Cr_2_O^2-^_7_ and the attraction between the ionic species and the surface of the adsorbent is large; thus, the adsorption is high. As the pH increases, the adsorption capacity drops as the charge on the surface of the adsorbent changes, and chromium species, which are more difficult to bind, form in the solution.

### 2.2. Cadmium

It is estimated that only 0.1 ppm of cadmium is present in Earth’s crust. It is usually found near sphalerite asa mineral, greenockite. Cadmium or its compounds are mainly used in battery manufacturing, electroplating, pigments, metal finishing, fertilizers, tanneries, and plastic manufacturing [11,25].

Cadmium, which occurs in industrial effluents, usually accumulates in soil. Soil contamination is also accelerated by the use of artificial phosphate fertilizers. Due to soil contamination, the most common cadmium exposure is through food intake. Inhalation of fine cadmium powder or vapor and ingestion of its soluble compounds is hazardous. Cadmium is toxic and carcinogenic even at low concentrations [11,25].

Cadmium exists in several different states, at pH levels below 7 as Cd^2+^, between pH 7 and 11 as Cd(OH)^+^, and above pH 11 as Cd(OH)_2_. Low pH values cause low adsorption of cadmium. If a higher adsorbent dose is added, the adsorption is enhanced because of the higher number of active sites. When the maximum dose of adsorbent is reached, the capacity starts to decrease again. Furthermore, it was observed that temperature also has an impact on adsorption; at higher temperatures, the adsorption of cadmium is reduced. Because copper has similar chemistry to cadmium, the adsorption capacity of cadmium decreased in the presence of copper [11].

Adsorption capacities of different adsorbents for cadmium removal are shown in Table 2.

**Table 2:** Cadmium (II) adsorption capacities/parameters.

Adsorbent	Adsorbent characterization	Adsorption conditions	Isotherm and kinetic models with calculated constants and adsorption capacities				Adsorption capacity	Ref
			Isotherm model	Calculated constants	Kinetic model	Calculated constants	Experimental	
Synthetic adsorbents								
CuO NP (copper oxide nanoparticles)	S_M_ = 220 m^2^/g, D_50_ = 33-160 nm	pH = 6, T = 25 ± 0.5 ◦C, c_0_ = 250 mg/L, m_ads_/V = 0.4 g/L	Langmuir	R^2^ = 0.9945 q_m_ = 84.75 mg/g	Pseudosecond	R^2^ = 0.989 q_e,cal_ = 131.33 mg/g		[3]
Manganese oxide	S_M_ = 84.9 m^2^/g, D_P50_ = 11.32 nm	pH = 6, T = 298 K, c_0_ = 100 mg/L, m_ads_/V = 1 g/L	Langmuir	R^2^ = 0.8889 q_m_ = 104.17 mg/g	Ppseudosecond	R^2^ = 0.9991 q_e,cal_ = 104.2 mg/g	R% = 98 % q_e,exp_ = 82.7 mg/g	[26]
FMWCNT (functionalized multiwall carbon nanotube)	S_M_ = 206.45 m^2^/g, V_P_ = 0.49 cm^3^/g, D_P_ = 96.27 Å	pH = 5, c_0_ = 10 mg/L, m_ads_/V = 1,0 g/100 mL, t_cont._ = 90 min	Langmuir Freundlich	R^2^ = 0.999 q_m_ = 83.33 mg/g R^2^ = 0.999	Pseudosecond	/	R% = 90 %	[27]
DWCNT/iron oxide composite	S_M_ = 127 m^2^/g, D_50_ = 10–30 nm, pH_pzc_ = 6	pH = 7, c_0_ = 20 mg/L, m_ads_/V = 50 mg/50 mL, t_cont._ = 50 min	/	/	/	/	R% = 95 % q_e,exp_ = 20.76 mg/g	[55]
A2/MWCNT (multiwall carbon nano tubes with polyamidoamine dendrimers)	S_M_ = 99.66 m^2^/g, D_P50_ = 21 nm, V_P_ = 0.584 cm^3^/g, pH_pzc_ = 5.92	pH = 6, c_0_ = 0.5 mg/L, m_ads_/V = 100 mg/L	Langmuir	R^2^ = 0.991 q_m_ = 44.18 mg/g	Pseudosecond	R^2^ = 0.993 q_e,cal_ = 2.338 mg/g	q_e,exp_ = 44.2 mg/g	[56]
Carboxyl modified Fe_3_O@SiO_2_ nanoparticles		pH = 6, c_0_ = 50 mg/L, m_ads_/V = 40 mg/20 mL, t_cont._ = 30 min	Langmuir	R^2^ = 0.999 q_m_ = 81.627 mg/g	/	/	R% = 71 %	[57]
M-CNT (modified carbon nano tubes)	d = 10–20 nm, L = 1–10 nm	pH = 7, c_0_ = 1 mg/L, m_ads_ = 50 mg, t_cont._ = 2 h	Langmuir	R^2^ = 0,993 q_m_ = 4.35 mg/g	Pseudosecond	R^2^ = 0.999	R% = 93 % q_e,exp_ = 2.02 mg/g	[58]
Natural organic/inorganic adsorbents								
KPP (Kaniar pod powder from Bauhinia purpurea)	S_M_ = 1.8 m^2^/g, D_P50_ = 22.6 nm, V_P_ = 0.01 cm^3^/g, pH_pzc_ = 4	pH = 5, T = 25 ◦C, c_0_ = 10 mg/L, m_ads_/V = 1 g/L	Langmuir	q_m_ = 11 mg/g	Pseudosecond	R^2^ = 0.99 q_e,cal_ = 10.31 mg/g	R% = 96 %, q_e,exp_ = 10.7 mg/g	[11]
MKPP (Magnetic Kaniar pod powder from Bauhinia purpurea)	S_M_ = 52 m^2^/g, D_P50_ = 15.3 nm, V_P_ = 0.2 cm^3^/g, pH_pzc_ = 5	pH = 5, T = 25 ◦C, c_0_ = 10 mg/L, m_ads_/V = 2 g/L	Langmuir	q_m_ = 4.8 mg/g	Pseudosecond	R^2^ = 0.98 q_e,cal_ = 3.8 mg/g	R% = 79 %, q_e,exp_ = 4.47 mg/g	[11]
CH-FeO nanocomposite (chitosaniron oxide nanocomposite)	D_50_ = 50 nm	pH = 3, T = 298 K, c_0_ = 10 mg/dm^3^, m_ads_/V = 0.05 g/20 dm^3^, t_cont._ = 30 min	Langmuir	R^2^ = 0.99	Pseudosecond	R^2^ = 0.99	q_e,exp_ = 201.84 mg/g	[28]
NTAA-LCM (nitrilotriacetic acid anhydride modified ligno cellulosic material)	S_M_ = 1.8006 m^2^/g, D_P_ = 8.9278 nm	pH = 4, T = 298 K, c_0_ = 200 mg/L, m_ads_ = 50 mg/50 mL	Langmuir	R^2^ = 0.998, q_m_ = 143.4 mg/g	Pseudosecond	R^2^ = 1 q_e,cal_ = 98.80 mg/g	q_e,exp_ = 98.81 mg/g	[31]
CMAC (Activated carbon from Cucumis melo peel)		pH = 6, c_0_ = 100 mg/L, m_ads_ = 250 mg/L, t_cont._ = 180 min	Elovich	R^2^ = 0.973	Pseudofirst	R^2^ = 0.993	R% = 97.96 %, q_e,exp_ = 7.23 mg/g	[34]
FGCX (aminobezoic grafted crosslinked chitosan)		pH = 6, T = 45 ◦C, c_0_ = 0,5 mmol/L, m_ads_/V = 6 g/L, t_cont._ = 60 min	Langmuir	R^2^ = 0.99 q_m_ = 1.82 mg/g	Pseudosecond	R^2^ = 0.99 q_e,cal_ = 3.52 mg/g	R% = 97.8 %	[35]
Dry biomass from Spirulina plantensis		pH = 8, T = 26 ◦C, c_0_ = 60 mg/L, m_ads_/V = 2 g/100 mL, t_cont._ = 90 min	Langmuir	R^2^ = 0.916			R% = 96.77 %	[59]
ZFA (zeolite fly ash)	S_M_ = 8.22 m^2^/g, D_P50_ = 29 Å	pH = 5, c_0_ = 50 mg/L, m_ads_ = 0,08 g/25 mL, t_cont._ = 7 h	Langmuir	R^2^ = 0.9919 q_m_ = 26.246 mg/g	Pseudosecond	R^2^ = 0.9964 q_e,cal_ = 16.583 mg/g	R% = 96 % q_e,exp_ = 14.431 mg/g	[60]
Mag-ligand	d = 30 nm	pH = 7, c_0_ = 10 mg/L, m_ads_ = 2 g/L	Langmuir	R^2^ = 0.951 q_m_ = 79.365 mg/g	Pseudosecond	R^2^ = 1 q_e,cal_ = 52.549 mg/g	R% = 97 %	[61]
Iron oxide nanoparticles with tanerine peel extract	D_50_ = 50 nm	pH = 4, m_ads_/V = 0.4 g/100 mL, t_cont._ = 90 min	Freundlich	R^2^ = 0.999, K_f_ = 1.789 mg/g	Pseudosecond	R^2^ = 0.999 q_e,cal_ = 10.9 mg/g	R% = 90 %	[62]
SHC (sunflower head carbon)	S_M_ = 2.5577 m^2^/g, D = 300 μm, pH_pzc_ = 3.8	pH = 6, T = 25 ± 1 ◦C, c_0_ = 100 mg/L, m_ads_/V = 20 g/L, t_cont._ = 120 min	Freundlich	K_f_ = 1.22 mg/g	Pseudosecond	R^2^ = 0.9978	R% = 84.7 % q_e,exp_ = 4.3 mg/g	[63]
SSC (sunflower stem carbon)	S_M_ = 2.6622 m^2^/g, D = 300 μm, pH_pzc_ = 4.2	pH = 6, T = 25 ± 1 ◦C, c_0_ = 100 mg/L, m_ads_/V = 20 g/L, t_cont._ =120 min	Freundlich	K_f_ =1.48 mg/g	Pseudosecond	R^2^ = 0.9981	R% = 87.1 % q_e,exp_ = 4.4 mg/g	[63]
GAC (granulated activated carbon)	S_M_ = 750 m^2^/g D = 0.3–2.4 nm, pH_pzc_ = 5.3	pH = 6.5, T = 24 ± 1 ◦C, c_0_ = 0.11 mg/L, t_cont._ = 24 h	Langmuir	R^2^ = 0.95 q_m_ = 2 mg/g	/	/	q_e,exp_ = 0.9 mg/g	[64]
CP (Peat mossderived biochar)		pH = 5, T = 20 ± 1 ◦C, c_0_ = 100 mg/L, m_ads_/V = 0.2/200 mL	Langmuir	R^2^ = 0.977 q_m_ = 39.8 mg/g	Second order	R^2^ = 0.999 q_e,cal_ = 29.5 mg/g	q_e,exp_ = 29.4 mg/g	[65]
BC-MnOx (biochar from rape straw impregnated with KMnO_4_)	S_M_ = 110.68 m^2^/g, V_P_ = 0.087 cm^3^/g	pH = 5, c_0_ = 20 mg/L, m_ads_/V = 1.25 g/L	Langmuir	R^2^ = 0.994 q_m_ = 81.1 mg/g	Pseudosecond	R^2^ = 0.998 q_e,cal_ = 7.288 mg/g	R% = 95%	[66]
B700-6 (biochar from orange peel with pyrolysis)	pH_pzc_ = 9.5	pH = 7, c_0_ = 100 mg/L, m_ads_/V = 0.1 g/50 mL	Langmuir	R^2^ = 0.87 q_m_ = 114.69 mg/g	Pseudosecond	R^2^ = 0.9996 q_e,cal_ = 32.68 mg/g	R% = 96.9% q_e,exp_ = 31.5 mg/g	[67]
Longan hull	S_M_ = 1.6 m^2^/g	pH = 5, c_0_ = 50 mg/L, m_ads_/V = 1 g/30 mL	Langmuir	R^2^ = 0.9954 q_m_ = 4.19 mg/g	Pseudosecond	/	R% = 93 %	[68]
PFP (palm fibers powder)		pH = 5, c_0_ = 100 mg/L, m_ads_/V = 1 g/100 mL, t_cont._ = 45 min	Freundlich	R^2^ = 0.990 K_f_ = 0.87 mg/g	Pseudo second	R^2^ = 0.998 q_e,cal_ = 6.430 mg/g	q_e,exp_ = 6.486 mg/g	[69]

S_M_ – specific surface area in a unit of mass, D_50_ – average particle size, D_P50_ – average pore size, V_P_ – pore volume, D_P_ – pore size, pH_pzc_ – point of zero charge, d – diameter, L – lenght, D – particle size, c_0_ – initial concentration, m_ads_/V – adsorbent dose per sample volume, t_cont._ - the time of contact od solution and adsorbent, m_ads_ – adsorbent dose, q_m_ – maximum adsorbent capacity (Langmuir), K_f_ – adsorption capacity (Freundlich), q_e,cal_ – calculated adsorption capacity, q_e,exp_ – experimentally determined adsorption capacity, R% – adsorption percent.

The best cadmium removal was achieved with the use of manganese oxide, with 98% cadmium removal [26]. Experimental data showed that the adsorption capacity of manganese oxide was 82.7 mg/g, and the maximum adsorption capacity calculated with the Langmuir model was 104.17 mg/g [26]. Functionalized multiwall carbon nanotubes (FMWCNT) [26] and copper oxide nanoparticles (CuO NP) [3] exhibited similar adsorption capacities, which were 83.33 mg/g [27] and 84.75 mg/g [3], respectively. Both adsorbents had similar surface areas of 206.45 m^2^ /g for FMWCNT [27] and 220 m^2^/g for CuO NP [3].

With natural organic and inorganic adsorbents, the best experimental results were reached with chitosan–iron oxide nanocomposite (CH-FeO nanocomposite), where the adsorption capacity of 201.84 mg/g was achieved experimentally [28]. This adsorbent was also effective in real wastewater samples, where 99.91% of cadmium was removed [28]. Chitosan consists of amino and hydroxyl groups that can bind heavy metals and form chelates. Chitosan itself is soluble under acidic conditions; with chitosan modification, a wider pH range can be obtained [29]. Furthermore, with modification procedures, adsorption properties and selectivity can be enhanced. The maximum adsorption capacity achieved with modified chitosan was 405 mg/g [30]. This value was attained with chitosan grafted with itaconic acid and crosslinked with glutaraldehyde (CS-g-IA(G). The results are not presented in this study, as Kyzas and Bikiaris have already conducted a detailed analysis in their review [30].

A maximum adsorption capacity of 143.4 mg/g was calculated for nitrilotriacetic acid anhydride-modified lignocellulosic material (NTAA-LCM) [31]. Experimental data showed that cadmium ions reacted with the carboxyl groups of adsorbents. It was established that sodium was exchanged with cadmium after adsorption took place. The adsorption process of cadmium onto NTAA-LCM occurred through surface chelation and ion exchange [31].

In general, it can be concluded that cadmium binding is not very successful at low pH values. The reason is that there are many H^+^ ions present in the aqueous solution which compete with Cd^2+^ for binding to the surface of the adsorbent. The adsorption of cadmium increases with increasing pH (pH from 4 to 8.5) as the surface of the adsorbent deprotonates and begins to attract cadmium ions. In accelerated adsorption, a mechaniS_M_ of cation exchange has been shown to occur in several cases.

### 2.3. Nickel

It is estimated that around 10% of the earth’s core is made of nickel. Much nickel is dissolved in water and accumulates in coal and oil. It occurs in millerite with sulphur, and in niccolite with arsenic. It can also be found in ores such as pentlandite. Nickel is a good conductor of heat and electricity. It is mainly used for alloys, as an additive for increasing the hardness of stainless steel, in the production of batteries, porcelain enameling, electroplating, dyeing, and pigments. A large amount of nickel is used in galvanization procedures because of its stability when in contact with atmospheric air [32].

Nickel only occurs in nature in low concentration levels. Nickel began to accumulate in the soil due to industry. Nickel present in soil can adsorb in sediments and become immobilized. In acidic soil nickel becomes mobile, which can result in it dissolving into groundwater. If groundwater is contaminated, exposure can happen with drinking. In addition, plants are subjected to nickel accumulation, exposing animals and humans to nickel through ingestion. It is an essential element in S_M_all quantities, but in high quantities, it can cause numerous negative consequences, such as lung, nose, or larynx cancer, lung emboliS_M_, asthma, chronic bronchitis, heart disorders, etc. [32,33].

Table 3 summarizes the studies on nickel adsorption. A review of synthetic adsorbents showed that the best nickel adsorption and maximum adsorbent capacity were achieved with amino-functionalized magnetic nanoadsorbent (Fe_3_O_4_-NH_2_) [2]. The percentage of nickel removed was above 96%, and the maximum capacity was 222.12 mg/g. In the study conducted with this adsorbent, removal of Cr (VI) was examined; high capacities were reached for Cr (VI) removal as well. In both cases, amino groups played a decisive role. The study of kinetic parameters confirmed the theory which suggested that adsorption might be achieved through chemical processes such as sharing or exchange of electrons. The difference between the two ions was in the optimal adsorption pH value. Nickel adsorption was more efficient at higher pH values than Cr (VI) adsorption. By increasing the pH value from 2 to 9, nickel removal increased from 46.21% to 93.03%, although it was also observed that nickel started to precipitate at a pH around 8.5. The study compared the results with other adsorbents such as Fe_3_O_4_-CNTs, Fe_3_O_4_ , and Fe_3_O_4_-GS with maximum adsorption capacities of 65.96 mg/g, 38.3 mg/g, and 158.5 mg/g, respectively. According to the comparative results, nanomagnetite functionalized with amino groups achieved the highest maximum adsorption [2].

**Table 3:** Nickel (II) adsorption capacities/parameters.

Adsorbent	Adsorbent characterization	Adsorption conditions	Isotherm and kinetic models with calculated constants and adsorption capacities				Adsorption capacity	Ref
			Isotherm model	Calculated constants	Kinetic model	Calculated constants	Experimental	
Synthetic adsorbents								
Fe_3_O_4_-NH_2_ (amino functionalized magnetic nanoadsorbent	D_50_ = 25 nm, pH_pzc_ = 5.8	pH = 6, T = 298 K, c_0_ = 5 mg/L, m_ads_/V = 0.05 g/50 mL	Langmuir	q_m_ = 222.12 mg/g	Pseudosecond	R^2^ = 0.994 q_e,cal_ = 25.97 mg/g	R% > 96 % q_e,exp_ = 25.12 mg/g	[2]
Carboxyl modified Fe_3_O@SiO_2_ nanoparticles		pH = 6, c_0_ = 50 mg/L, m_ads_/V = 50 mg/20 mL, t_cont._ = 30 min	Langmuir	R^2^ = 0.989 q_m_ = 63.995 mg/g			R% = 65 %	[57]
Activated carbon coated Al_2_O_3_ nanoparticles	D_50_ = 50 nm	c_0_ = 300 mg/L, m_ads_/V = 20 mg/50 mL, t_cont._ = 180 min	/	/	/	/	R% = 82 %	[70]
GO (Graphene oxide)	W = 0.77–2.94 nm	pH = 7, c_0_ = 25 mg/L, m_ads_/V = 20 mg/200 mL, t_cont._ = 320 min	Langmuir	R^2^ = 0.9993 q_m_ = 35.6 mg/g	Pseudosecond	q_e,cal_ = 32.4 mg/g	q_e,exp_ = 31.7 mg/g	[71]
Fe@G (magnetic Fe@graphite nanocomposite)	S_M_ = 47 m^2^/g, V_P_ = 17.7 cm^3^/g, r_p_ = 0.016 nm, pH_pzc_ = 7.7	pH = 8.2, T = 20 ◦C, c_0_ = 20 mg/L	Langmuir	R^2^ = 0.998 q_m_ = 9.2 mg/g	Pseudosecond	R^2^ = 0.999 q_e,cal_ = 8.95 mg/g	q_e,exp_ = 9.33 mg/g	[72]
Natural organic/inorganic adsorbents								
Fe_3_O_4_/talc nanocomposite	S_M_ = 37.079 m^2^/g	pH = 7, c_0_ = 92 mg/L, m_ads_ = 0.12 g, t_cont._ = 120 s	Langmuir	R^2^ = 0.9772 q_m_ = 33.33 mg/g	Pseudosecond	R^2^ = 0.9812	R% = 50.23 %	[9]
LNC/MMT (Lignocellulose/ montmorillonite nanocomposite)		pH = 6.8, T = 70 ◦C, c_0_ = 0.0032 mol/L, m_ads_/V = 0.1 g/50 mL, t_cont._ = 40 min	Langmuir	R^2^ = 0.9989 q_m_ = 94.86 mg/g	Pseudosecond	R^2^ = 0.998 q_e,cal_ = 87.72 mg/g	q_e,exp_ = 94.86 mg/g	[12]
CH-FeO nanocomposite (chitosaniron oxide nanocomposite)	D_50_ = 50 nm	pH = 3, T = 298 K, c_0_ = 10 mg/dm^3^, m_ads_/V = 0.05 g/20 dm^3^, t_cont._ = 1800 s	Langmuir	R^2^ = 0.99 q_m_ = 3.29 mg/g	Pseudosecond	R^2^ = 0.99 q_e,cal_ = 3.772 mg/g	q_e,exp_ = 57.86 mg/g	[28]
CMAC (Activated carbon from Cucumis melo peel)		pH = 6, c_0_ = 100 mg/L, m_ads_/V = 250 mg/L, t_cont._ = 180 min	Elovich	R^2^ = 0.976	Pseudofirst	R = 0.973	R% = 98.78 %q e,exp = 5.43 mg/g	[34]
FGCX (aminobezoic grafted crosslinked chitosan)		pH = 7, T = 45 ◦C, c_0_ = 0.5 mmol/L, m_ads_/V = 6 g/L, t_cont._ = 60 min	Langmuir	R^2^ = 0.99 q_m_ = 2.8 mmol/g	Pseudosecond	R^2^ = 1.0 q_e,cal_ = 3.12 mmol/g	R% = 98.6 %	[35]
GAC (granulated activated carbon)	S_M_ = 750 m^2^/g D = 0.3–2.4 nm, pH_pzc_ = 5.3	pH = 6.5, T = 24 ± 1 ◦C, c_0_ = 0.12 mg/L, t_cont._ = 24 h	Langmuir	R^2^ = 0.98 q_m_ = 1.8 mg/g	/	/	q_e,exp_ = 1.3 mg/g	[64]
Longan hull	S_M_ = 1.6 m^2^/g	pH = 5, c_0_ = 50 mg/L, m_ads_/V = 1 g/30 mL	Langmuir	R^2^ = 0.9696 q_m_ = 3.96 mg/g	Pseudosecond	/	R% = 90 %	[68]
PFP (palm fibers powder)		pH = 5, c_0_ = 100 mg/L, m_ads_/V = 1 g/100 mL, tcontact = 45 min	Freundlich	R^2^ = 0.937 K_f_ = 5.058 mg/g	Pseudosecond	R^2^ = 0.996 q_e,cal_ = 3.736 mg/g	q_e,exp_ = 3.746 mg/g	[69]
Chitosan		pH = 5, T = 25 ± 1 ◦C, c_0_ = 100 mg/L, m_ads_/V = 5 g/100 mL	Langmuir	R^2^ = 0.997 q_m_ = 52.6 mg/g	Pseudosecond	R^2^ = 0.997 q_e,cal_ = 10.63 mg/g	q_e,exp_ = 9.6 mg/g	[73]
AC (activated carbon from sewage sludge)	S_M_ = 132.7 m^2^/g, V_P_ = 0.23 cm^3^/g	pH = 8, T = 55 ◦C, c_0_ = 50 mg/L, m_ads_/V = 4 g/L	Langmuir	R^2^ = 0.9655 q_m_ = 11.52 mg/g	Pseudosecond	R^2^ = 0.999 q_e,cal_ = 9.8078 mg/g	q_e,exp_ = 9.71 mg/g	[74]
ONF/KOH (old newspaper fibers treated with KOH)	D_50_ = 53.87 μm	pH = 6, T = 25 ◦C, c_0_ = 100 mg/L, m_ads_/V = 4 g/L	Langmuir	R^2^ = 0.9825 q_m_ = 20.2 mg/g	Pseudosecond	R^2^ = 0.9998 q_e,cal_ = 18.31 mg/g	R% = 88 %	[75]
GAC (activated carbon from Gyricidia	D = 45–180 μm	pH = 7, c_0_ = 50 mg/L, m_ads_/V = 0.4 g/100 mL	Langmuir	R^2^ = 0.879 q_m_ = 24.39 mg/g	/	/	R% = 97.3 % q_e,exp_ = 12.1 mg/g	[76]
Sawdust)		pH = 6, c_0_ = 100 mg/L, m_ads_/V = 1.75 g/100 mL	Langmuir	R^2^ = 0.982 q_m_ = 12.5 mg/g	Pseudosecond	R^2^ = 0.942 q_e,cal_ = 5.62 mg/g	R% = 94.6 % q_e,exp_ = 5.41 mg/g	[77]
Chitosan/kenaf fiber bio-composite		pH = 5, c_0_ = 200 mg/L, m_ads_/V = 1 g/100 mL	Langmuir	R^2^ = 0.8916 q_m_ = 70.55 mg/g	Pseudosecond	R^2^ = 0.999 q_e,cal_ = 10.67 mg/g	R% = 89 %	[78]

D_50_ – average particle size, pH_pzc_ – point of zero charge, W – thickness, S_M_ – specific surface area in a unit of mass, V_P_ – pore volume, D – particle size, r_p_ – radius, c_0_ – initial concentration, m_ads_/V – adsorbent dose per sample volume, t_cont._ - the time of contact od solution and adsorbent, q_m_ – maximum adsorbent capacity (Langmuir), K_f_ – adsorption capacity (Freundlich), q_e,cal_ – calculated adsorption capacity, q_e,exp_ – experimentally determined adsorption capacity, R% – adsorption percent.

Furthermore, natural organic and inorganic adsorbents also showed good experimental results. Removal rates of 98.78% [34] and 98.6% [35] were attained with activated carbon from Cucumis melo peel (CMAC) [34] and aminobenzoic grafted crosslinked chitosan (FGCX) [35], respectively.

Activated carbon (AC) is a widely used adsorbent. Its main feature is a porous structure which enables a large surface area. The surface area of AC can range from 300 to 4000 m^2^ /g. Additional surface functional groups can greatly improve adsorption [6]. According to the analysis of the data that was obtained for CMAC, it could be concluded that the adsorption process can be attributed to a chemical reaction with functional groups on the surface [34].

Chitosan itself is a suitable adsorbent because it contains amine and hydroxyl groups [35]. Its disadvantage is that it is soluble at low pH values. Crosslinking helps overcome this disadvantage [29]; however, some studieshave noted that crosslinking can reduce the overall adsorption capacity of an adsorbent. Furthermore, grafting allows the formation of functional derivatives with more established adsorption sites, which compensates for possible diminishment in capacity on account of crosslinking. The study of Igberase indicates that the binding of metal ions can occur through chemisorption, electrostatic attraction, or ion exchange [35].

A maximum adsorption capacity of 94.86 mg/g was achieved with lignocellulose/montmorillonite nanocomposite (LNC/MMT) [12]. By analyzing the adsorption procedure, it was shown that the main mechaniS_M_ of adsorption was chemical adsorption. Additional adsorbent studies showed that hydroxyl and carboxyl groups were involved in adsorption process. Furthermore, in the results of a desorption study, LNC/MMT showed stability after desorption, indicating that the adsorbent is stable and has possible applications in industry [12].

The most successful results for nickel adsorption come from binding to amino groups at pH values between 6 and 8. At lower pH levels, many H+ ions that are present in solution are protonating –NH_2_ to NH+3 . This causes a repulsive force between the positive surface and the nickel ions. When pH is increased, deprotonation of amino groups occurs, thus allowing nickel binding, which is reflected in higher adsorption.

## 3. Discussion

Generally good results for chromium removal were obtained in an acidic environment that extended from pH value 1 to pH value 4. Low pH values can protonate the adsorbent surface. A positively charged surface is optimal for the removal of Cr species present in solution at this pH. The binding expires with electrostatic attraction. Experiments were conducted at pH value 7 in only one study out of all of the studies that we reviewed regarding chromium removal. The adsorbent used at these higher pH values was red mud modified by lanthanum (La-RM) [36]; the experimentally achieved adsorption capacity was 17.35 mg/g. The optimal pH value for Cr (VI) adsorption was adsorbent-dependent. Furthermore, a review of various studies has shown that specific surface area is not a determining factor for the adsorption process. It has also been shown that amino and hydroxyl groups can be strongly involved in the adsorption process and are usually responsible for enhancing chromium adsorption. Furthermore, the results of natural organic and inorganic adsorbents showed the great potential of adsorbents functionalized with amino and hydroxyl groups, and of graphene sand composite.

Cadmium adsorption was achieved with acidic and neutral solutions. The pH values of the various experiments ranged from 3 to 8, although the best results were obtained at slightly higher pH values, e.g., 6. The reason for the better adsorption at higher pHs lies in the deprotonation of the surface of the adsorbent, which in turn allows electrostatic attraction of cadmium. In addition, cadmium removal studies have shown that there is no correlation between specific surface area and adsorption. More accurate information about surface properties (e.g., micropore volume, total pore volume, macropore volume, pore diameter) would be required to properly evaluate the effect of a given surface area on adsorption.

Due to similar properties of heavy metals and similar removal mechanisms, nickel removal studies have shown similar results to those of chromium and cadmium removal. Amino and hydroxyl groups show potential for nickel adsorption. Moreover, chitosan and graphene showed high potential for removal of all examined ions. It was also seen that nickel binding performed more successfully in a less acidic environment between pH values 6 and 8, as the deprotonated surface of the adsorbent is important for electrostatic attraction. As the pH of the solution increased, nickel adsorption increased.

Comparing metal removal, natural adsorbents performed better in all 3 cases.

## References

[1] (2010). Best avaliabe Techniques (BAT) Reference document for common waste water and waste gas treatment/management systems in the shemical sector. JRC Science for Policy Report. Industrial Emissions Directive.

[2] (2016). One-pot synthesis, characterization and adsorption studies of amine-functionalizedmmagnetite nanoparticles for removalmof Cr(VI) and Ni(II) ions from aqueousmsolution: kinetic, isotherm and thermodynamic studies. Journal of environmental Healts Science and Engineering.

[3] (2015). Metal oxide nano-particles as an adsorbent for removal of heavy metals. Journal of Advanced Chemical Engineering.

[4] (2017). Batch adsorption of Cr(VI) ions on zeolite and agroindustrial waste. Chemical and Biochemical Engineering Quarterly.

[5] (2016). Facile synthesis of zero valent iron magnetic biochar composites for Pb(II) removal from the aqueous medium. Alexandria Engineering Journal.

[6] (2003). Adsorbents: fundamentals and applications. Hoboken, NJ, USA: John Wiley and Sons Inc..

[7] (2001). Unit operations of chemical engineering. New York, NY, USA: McGeaw-Hill Inc..

[8] (2018). Removal of Pb(II) and Cr(VI) from aqueous solutions using the prepared porous adsorbent-supportedm Fe/Ni nanoparticles. RSC Advances.

[9] (2014). Rapid adsorption of heavy metals by Fe$_{3}$O$_{4}$/Talc nanocomposite and optimization study using response surface methodology. International Journal of Molecular Sciences.

[10] (2015). Facile synthesis of polypyrrole decorated reduced graphene oxide-Fe$_{3}$O$_{4}$ magnetic composites and its application for the Cr(VI) removal. Engineering Journal.

[11] (2017). Cadmium and lead remediation using magnetic and non-magnetic sustainable biosorbents derived from Bauhinia purpurea pods. RSC Advances.

[12] (2015). Adsorption and desorption of nickel(II) ions from aqueous solution by a lignocellulose/ montmorillonite nanocomposite. PLOS ONE.

[13] (2016). A study on effects of pH, adsorbent dosage, time, initial concentration and adsorption isotherm study for the removal of hexavalent chromium (Cr(VI)) from wastewater by magnetite nanoparticles. Procedia Technology.

[14] (2015). Sorption and desorption of Cr(VI) ions from water by biochars in different environmental conditions. Environmental Science and Pollution Research.

[15] (2015). Removal of Chromium(VI) from aqueous solutions using Fe$_{3}$O$_{4}$ magnetic polymer microspheres functionalized with amino groups. Materials.

[16] (2018). Adsorption effectivity test of Andisols Clay-Zeolite (ACZ) composite as chromium hexavalent (Cr(VI)) ion adsorbent. Science and Engineering.

[17] (2015). Adsorption of Cr(VI) in wastewater using magnetic multi-wall carbon nanotubes. Water science and Engineering.

[18] (2014). Sorption of Cr(III) and Cr(VI) to high and low pressure synthetic nano-magnetite (Fe$_{3}$O$_{4})$ particles. Chemical Engineering Journal.

[19] (2016). Advanced materials letters chromium(VI) ions adsorption onto barium hexaferrite magnetic nano-adsorbent. Advanced Materials Letters.

[20] (2015). Effective adsorption of chromium(VI)/Cr(III) from aqueous solution using ionic liquid functionalized multiwalled carbon nanotubes as a super sorbent. Journal of Materials Chemistry A.

[21] (2018). Adsorption of hexavalent chromium from aqueous solution by activated carbon prepared from almond shell: Kinetics, equilibrium and thermodynamics study. Journal of Water Supply: Research and Technology.

[22] (2017). Adsorption of Cr(VI) from aqueous phase by high surface area activated carbon prepared by chemical activation with ZnCl2. Process Safety and Environmental Protection.

[23] (2016). Effect of acid modification on adsorption of hexavalent chromium (Cr(VI)) from aqueous solution by activated carbon and carbon nanotubes. Desalination and Water Treatment.

[24] (2015). Green synthesis of graphene sand composite (GSC) as novel adsorbent for efficient removal of Cr(VI) ions from aqueous solution. Journal of Water Process Engineering.

[25] (2009). Nano-adsorbents for the removal of metallic pollutants from water and wastewater. Environmental Technology.

[26] (2017). The Adsorption of Cd(II) on manganese oxide investigated by batch and modeling techniques. International Journal of Environmental Research and Public Health.

[27] (2015). Comparative kinetic study of functionalized carbon nanotubes and magnetic biochar for removal of Cd2+ ions from wastewater. Korean Journal of Chemical Engineering.

[28] (2017). Synthesized chitosan iron oxide nanocomposite and shrimp shell in removal of nickel, cadmium and lead from aqueous solution. Global Journal of Environmental Science and Management.

[29] (2016). Heavy metal removal from wastewater using various adsorbents: a review. Journal of Water Reuse and Desalination.

[30] (2015). Recent modifications of chitosan for adsorption applications: a critical and systematic review. Marine Drugs.

[31] (2015). Removal of cadmium and lead from aqueous solutions using nitrilotriacetic acid anhydride modified ligno-cellulosic material. RSC Advances.

[32] (2012). IARC working group on the evaluation of carcinogenic risk to humans. Arsenic, metals, fibers and dusts. Lyon, France: International Agency for Research on Cancer.

[33] (2011). Nickel: an overview of uptake, essentiality and toxicity in plants. Bulletin of Environmental Contamination and Toxicology.

[34] (2018). Kinetic study on adsorption of Cr(VI), Ni(II), Cd(II) and Pb(II) ions from aqueous solutions using activated carbon prepared from Cucumis melo peel. Applied Water Science.

[35] (2017). The adsorption of Pb, Zn, Cu, Ni, and Cd by modified ligand in a single component aqueous solution: equilibrium, kinetic, thermodynamic, and desorption studies. International Journal of Analytical Chemistry.

[36] (2016). Cr(VI) adsorptionon on red mud modified by lanthanum: performance, kinetics and mechanisms. PLOS ONE.

[37] (2017). Synthesis, characterization and Cr(VI) adsorption properties of modified magnetite nanoparticles. Acta Physica Polonica A.

[38] (2017). Applications of CTAB modified magnetic nanoparticles for removal of chromium(VI) from contaminated water. Journal of Advanced Research.

[39] (2017). Polyaniline nanofibers and nanocomposites: preparation, characterization, and application for Cr(VI) and phosphate ions removal from aqueous solution. Arabian Journal of Chemistry.

[40] (2018). Preparation and application synthesis of magnetic nanocomposite using waste toner for the removal of Cr(VI). RSC Advances.

[41] (2018). A hydrothermal synthesis of Fe3O4@C hybrid nanoparticle and magnetic adsorptive performance to remove heavy metal ions in aqueous solution. Nanoscale Research Letters.

[42] (2014). and characterization of barium ferrite containing magnetic affinity microbeads and isotherm analysis of Cr(VI) ions adsorption from aqueous solutions. Journal of Biological Chemistry.

[43] (2016). Adsorption of hexavalent chromium onto activated carbon derived from Leucaena leucocephala waste sawdust: kinetics, equilibrium and thermodynamics. International Journal of Environmental Science and Technology.

[44] (2017). Simultaneous reductive and sorptive removal of Cr(VI) by activated carbon supported b-FeOOH. RSC Advances.

[45] (2018). Reduction and removal of chromium VI in water by powdered activated carbon. Materials.

[46] (2016). Chromium(VI) adsorption from aqueous solution by prepared biochar from Onopordom Heteracanthom. International Journal of Environmental Science and Technology.

[47] (2016). Fabrication of chitosan-magnetite nanocomposite strip for chromium removal. Applied Nanoscience.

[48] (2018). Potential use of green algae as a biosorbent for hexavalent chromium removal from aqueous solutions. Saudi Journal of Biological Sciences.

[49] (2016). Effects of Cu(II) on the adsorption behaviors of Cr(III) and Cr(VI) onto kaolin. Journal of Chemistry.

[50] (2016). Investigating selective removal of Cr(VI) and zinc ions from aqueous media by mechanical-chemical activated red mud. Iranian Journal of Materials Science and Engineering.

[51] (2014). Hexavalent chromium removal from water using heat-acid activated red mud. Open Journal of Applied Sciences.

[52] (2014). Removal of hexavalent chromium from aqueous solution by granular and powdered Peganum Harmala. Applied Surface Science.

[53] (2015). Isotherm modelling, kinetic study and optimization of batch parameters using response surface methodology for effective removal of Cr(VI) using fungal biomass. PLOS ONE.

[54] (2015). Biosorption of Cr(VI) by ceratocystis paradoxa MSR2 using isotherm modelling, kinetic study and optimization of batch parameters using response surface methodology. PLOS ONE.

[55] (2017). Adsorption of cadmium ions from water on doublewalled carbon nanotubes/iron oxide composite. Chemistry Journal of Moldova, General, Industrial and Ecological Chemistry.

[56] (2017). Removal of heavy metals from water using multistage functionalized multiwall carbon nanotubes. Journal of the Serbian Chemical Society.

[57] (2017). Synthesis of carboxyl-modified Fe3O4$@SiO2 nanoparticles and their utilization for the remediation of cadmium and nickel from aqueous solution. Journal of the Chilean Chemical Society.

[58] (2015). Adsorptive removal of cadmium(II) ions from liquid phase using acid modified carbon-based adsorbents. Journal of Molecular Liquids.

[59] (2015). Adsorptive removal of cadmium ions by Spirulina platensis dry biomass. Saudi Journal of Biological Sciences.

[60] (2015). Study of the adsorption of Cd (II) from aqueous solution using zeolite-based geopolymer, synthesized from coal fly ash; kinetic, isotherm and thermodynamic studies. Arabian Journal of Chemistry.

[61] (2015). EDTA functionalized magnetic nanoparticle sorbents for cadmium and lead contaminated water treatment. Water Research.

[62] (2015). Cadmium removal from aqueous solution by green synthesis iron oxide nanoparticles with tangerine peel extract. Journal of Environmental Healts Science and Engineering.

[63] (2015). Cadmium removal from wastewater using carbonaceous adsorbents prepared from sunflower waste. International Journal of Environmental Research.

[64] (2015). Effects of humic acid and suspended solids on the removal of heavy metals from water by adsorption onto granular activated carbon. International Journal of Environmental Research and Public Health.

[65] (2015). Comparison of heavy metal adsorption by peat moss and peat moss-derived biochar produced under different carbonization conditions. Water, Air and Soil Pollution.

[66] (2017). Adsorption of Cd(II) from aqueous solutions by rape straw biochar derived from different modification processes. Chemosphere.

[67] (2016). Effect of pyrolysis temperatures and times on the adsorption of cadmium onto orange peel derived biochar. Waste Management and Research.

[68] (2018). Adsorptive removal of Ni2+ and Cd2+ from wastewater using a green longan hull adsorbent. Adsorption Science and Technology.

[69] (2017). Removal of nickel (II) and cadmium (II) ions from wastewater by palm fibers. Scientific Study and Research Chemistry and Chemical Engineering.

[70] (2017). Synthesis and characterization of activated carbon coated alumina as nano adsorbent. Materials Today.

[71] (2017). Equilibrium and kinetics studies for the adsorption of Ni2+ and Fe3+ ions from aqueous solution by graphene oxide. Polish Journal of Chemical Technology.

[72] (2016). Adsorption of Ni2+ from aqueous solution by magnetic Fe@graphite nanocomposite. Polish Journal of Chemical Technology.

[73] (2016). Removal of Ni(II) and Zn(II) from aqueous solutions using chitosan. Archives of Hygiene Sciences.

[74] (2018). Nickel (II) adsorption from aqueous solutions by physico-chemically modified sewage sludge. Iranian Journal of Chemistry and Chemical Engineering.

[75] (2016). KirosY. International Journal of Environmental Research.

[76] (2015). Adsorption of Ni (II) ion from metal solution using natural adsorbents international. Journal of Emerging Trends in Engineering and Development.

[77] (2017). Nickel ions removal from aqueous solutions using sawdust as adsorbent: equilibrium, kinetic and thermodynamic studies. Journal of Engineering and sustainable Development.

[78] (2015). Adsorption study: removal of nickel ions using kenaf fiber/chitosan biosorbent. Journal of Chemical and Pharmaceutical Research.

